# Dyslipidemia Is Negatively Associated With the Cumulative Live-Birth Rate in Patients Without PCOS Following IVF/ICSI

**DOI:** 10.3389/fphys.2021.713356

**Published:** 2021-08-13

**Authors:** Zhenteng Liu, Jianxiang Cong, Xuemei Liu, Huishan Zhao, Shoucui Lai, Shunzhi He, Hongchu Bao

**Affiliations:** Department of Reproductive Medicine, Yantai Yuhuangding Hospital Affiliated to Qingdao University, Yantai, China

**Keywords:** dyslipidemia, assisted reproductive technology, controlled ovarian hyperstimulation, obesity, cumulative live-birth rates

## Abstract

**Objective:** To evaluate the effect of dyslipidemia on the cumulative live-birth rate (cLBR) in patients without polycystic ovary syndrome (PCOS) undergoing *in vitro* fertilization/intracytoplasmic sperm injection–embryo transfer (IVF/ICSI–ET) cycles.

**Methods:** A total of 1,132 patients from the Yantai Yuhuangding Hospital Affiliated to Qingdao University from January 2016 to December 2017 were retrospectively included. The subjects were distributed into two groups based on their lipid profiles, namely, dyslipidemia group (*n* = 195) and control group (*n* = 937). The clinical and laboratory parameters of the two groups were analyzed, and a multivariate logistic regression analysis of the cLBR was conducted. In addition, subgroup analysis was carried out to avoid deviation according to the body mass index (BMI).

**Results:** Patients with dyslipidemia had significantly greater BMI and longer duration of infertility, as well as lower antral follicle count and basal follicle-stimulating hormone level compared with patients without dyslipidemia. Stratified analysis showed that dyslipidemia was associated with a significantly higher total gonadotrophin dosage required for ovarian stimulation as well as lower number of oocytes retrieved, independent of obesity. The live-birth rate in fresh cycle and cLBR were higher in the control group, although the difference between the groups was not significant (54.9% vs. 47.3%, *p* = 0.116; 67.6% vs. 62.1%, *p* = 0.138). However, multivariate logistic regression analysis adjusting for potential confounders showed that dyslipidemia was negatively associated with cLBR (OR, 0.702, 95% CI, 0.533–0.881, *p* = 0.044).

**Conclusion:** Our findings demonstrate for the first time that dyslipidemia has a deleterious impact on cLBR, independent of obesity, in non-PCOS population considered to have good prognosis. Assessment of serum lipid profiles as well as the provision of nutritional counseling is essential for increasing successful outcomes in assisted reproductive techniques.

## Introduction

Recently, there has been a significant increase in the incidences of infertility and metabolic disorders, such as dyslipidemia and obesity, which has been associated with unhealthy lifestyles and poor living conditions (Broughton and Moley, 2017). To date, individual and combined contributions of hormonal, metabolic, and environmental factors on reproductive disorders in women of childbearing age remain unclear (Hohos and Skaznik-Wikiel, 2017). Notably, excessive calorie intake, particularly a high-fat diet, is a risk factor for both dyslipidemia and reproductive dysfunction (Lie et al., 2013; Newell-Fugate et al., 2014).

Dyslipidemia is clinically defined as abnormalities in one or more types of plasma lipids, such as elevated total cholesterol (TC), low-density lipoprotein cholesterol (LDL-C), and triglyceride (TG) concentrations, as well as reduced high-density lipoprotein cholesterol (HDL-C) levels (Joint Committee for Guideline Revision, 2018). In China, the prevalence of dyslipidemia in adults increased from 18.6% in 2002 to 40.4% in 2012 (He et al., 2014; Joint Committee for Guideline Revision, 2018), posing a major public health problem. Although abnormal lipid metabolism is frequently accompanied by obesity, dyslipidemia can also be found in infertile patients without obesity (Legro et al., 2001; Wild et al., 2011).

Several studies using mouse models have shown that chronic consumption of high-fat diet (HFD) results in metabolic and reproductive disorders independent of obesity. Specifically, mice on HFD ovulated less often (Hohos et al., 2020), had diminished ovarian reserve, increased ovarian inflammation (Skaznik-Wikiel et al., 2016), altered ovarian gene expression (Hohos et al., 2018), and had oocyte-specific defects (Wu et al., 2010; Reynolds et al., 2015) and lower fertility rates (Skaznik-Wikiel et al., 2016), regardless of the obesity phenotype. Interestingly, the HFD-induced oocyte-specific defects, such as increased lipid deposition, were not reversed by switching to a standard low-fat diet, despite reversal of metabolic parameters (Reynolds et al., 2015). Recently, Pugh et al. suggested there was a 19–36% reduction in the likelihood of spontaneous pregnancy per cycle for women with irregular serum lipid conditions without a diagnosis of infertility (Pugh et al., 2017). Therefore, it can be inferred that dyslipidemia may be an independent risk factor for female sterility. Understanding the effect of dyslipidemia on reproductive physiology is important for developing strategies to improve pregnancy outcomes.

In the last few decades, most studies have generally focused on the effect of metabolic disorders, such as obesity (Broughton and Moley, 2017) and polycystic ovary syndrome (PCOS) (Kollmann et al., 2016), on the reproductive function and outcomes. However, almost no published clinical study investigated the impact of abnormal maternal lipid levels on the outcomes of assisted reproductive treatments independent of obesity. Thus, we performed the current study to provide insights into the effects of dyslipidemia on IVF/ICSI outcomes, such as cumulative live-birth rate (cLBR), in the general non-PCOS population.

## Materials and Methods

### Study Design and Subjects

This was a retrospective cohort study. All women attending the Department of Reproductive Medicine at the Yantai Yuhuangding Hospital Affiliated to Qingdao University in the People’s Republic of China from January 2016 to December 2017 were screened for eligibility. The study received ethical approval from the Ethics Committee of Yantai Yuhuangding Hospital, and all the protocols were conducted in accordance with relevant guidelines and regulations. The need for informed consent for this study was waived due to the retrospective nature of the study.

All patients aged 20–38 years, who had a normal ovarian reserve which is generally defined as the basal antral follicular count of more than seven (Christianson et al., 2015). Our criteria for women without PCOS are that all women had a regular menstrual cycle and none had polycystic ovaries on ultrasound. Women with a diagnosis of PCOS based on the Rotterdam criteria (two out of the triad of oligo-amenorrhea, biological or clinical hyperandrogenism and multi-follicular appearance of ovaries on transvaginal ultrasound scan; The Rotterdam ESHRE/ASRM—Sponsored PCOS Consensus Workshop Group, 2004) were excluded from the study (Rotterdam, 2004). To avoid bias arising from the use of different ovarian stimulation protocols, only women who received their first ovarian stimulation cycle, using luteal phase gonadotrophin-releasing hormone (GnRH) agonist protocol or GnRH antagonist, were included in the study. Each patient was included only once in the analysis.

The exclusion criteria included the following: (1) history of chemotherapy, radiotherapy, or ovarian surgery; (2) endometriosis; (3) uterine abnormality; (4) indication for preimplantation genetic diagnosis or preimplantation genetic screening; (5) history of recurrent pregnancy loss; and (6) other medical conditions that are contraindicated in assisted reproductive technology and/or pregnancy.

Blood samples were collected between day 2 and day 4 of the menstrual cycle before the IVF/ICSI protocol after an overnight fast of at least 8 h. The serum concentrations of lipoproteins, including total cholesterol (TC), LDL-C, HDL-C, and triglyceride (TG), were then evaluated by an automatic biochemical analyzer (Cardiocheck PA). All eligible individuals who had a known fresh cycle outcome (live-birth or not) were included in our study. Individuals who did not deliver a live baby in the fresh cycle were followed for at least 2 years (irrespective of the date of study entry), to estimate the outcomes of the frozen-thawed cycles. The study participants were divided into two groups based on their lipid profiles. According to the 2016 Chinese guidelines for the management of dyslipidemia in adults (Joint Committee for Guideline Revision, 2018), participants were defined as having dyslipidemia if they had one or more of the following conditions: TC ≥ 6.22 mmol/L (240 mg/dl), LDL-C ≥ 4.14mmol/L (160 mg/dl), HDL-C ≤ 1.04mmol/L (40 mg/dl), or TG ≥ 2.26mmol/L (200 mg/dl).

### Protocols of IVF/ICSI

In our center, the standard GnRH agonist long protocol or GnRH antagonist protocol is used in patients with normal ovarian function. Briefly, for the GnRH agonist protocol, the women received a daily subcutaneous injection of 0.03–0.05 mg GnRH agonist, starting in the luteal phase of the previous cycle until the day of human chorionic gonadotropin (hCG) administration. After stimulation with GnRH for 14–21 days and satisfactory pituitary desensitization had been achieved (serum estradiol level lower than 50 pg./ml, luteinizing hormone <5 IU/L, size of follicular <10 mm, and endometrial thickness <5 mm), recombinant follicle-stimulating hormone (rFSH) was administered in concentrations ranging from 150 to 225 IU/day. This was adjusted according to follicular development monitored using transvaginal ultrasonography and assessment of the serum concentrations of sex hormones. For the flexible GnRH antagonist protocol, rFSH was started on days 2–3 of the menstrual cycle, and the doses were modulated based on the ovarian response of the patients. Human menopausal gonadotropin was co-administered at the discretion of the physicians. Ganirelix (0.25 mg) was administered daily when the lead follicle >12 mm, up to and including the day of hCG administration. Urinary hCG (4,000–10,000 IU) was administered when at least two dominant follicles measured 18 mm or more in diameter. Oocyte retrieval was performed 34–36 h later.

Based on sperm quality, oocytes were fertilized through conventional IVF or ICSI or via IVF/ICSI. The embryos were cultured up to day 3 or day 5/6. Embryo culture was carried out using sequential culture medium (G-Series, Vitrolife, Sweden). Embryo assessment, including cleavage-stage embryo assessment and blastocyst assessment, was performed based on the recommendation of the Istanbul consensus workshop (Alpha Scientists in Reproductive Medicine and E.S.I.G.E, 2011). Redundant embryos (or all embryos in case of a freeze-all policy) were vitrified on day 3 or day 5/6 by closed vitrification using closed blastocyst vitrification high security straws (Cryo Bio System, Paris, France) combined with dimethyl sulfoxide and ethylene glycol bis (succinimidyl succinate) as the cryoprotectants (Irvine Scientific Freeze Kit, Canada). Frozen-thawed embryos were transferred after either the natural or mild stimulation cycle of the patient, or artificial preparation with or without GnRH downregulation. Luteal-phase support (oral dydrogesterone or vaginal progesterone) was scheduled after oocyte retrieval in fresh cycle or at endometrial transformation day in frozen-ET cycle and was continued until 10 weeks of gestation.

### Main Outcome Measures

Our primary outcome was the cLBR, which was defined as the delivery of a live-born infant (>24 weeks of gestation) in the fresh or in the subsequent frozen-thawed cycles in relation to the number of oocytes retrieved within two years (Polyzos et al., 2018). s was considered in the analysis. The secondary outcome was live-birth after the fresh IVF/ICSI cycle only. Miscarriage was defined as spontaneous loss of a clinical pregnancy before completion of 24 gestational weeks. We defined OHSS according to the OHSS prevention and treatment guideline of the American Society for Reproductive Medicine (Practice Committee of the American Society for Reproductive Medicine, 2016).

### Statistical Analysis

All analyses were carried out using IBM SPSS Statistics (version 26.0; IBM, Inc.). Normal distribution of quantity variables was tested using the Shapiro-Wilk test. Continuous variables were presented as means ± standard deviations (SDs), which were compared using the Student’s *t*-test (normally distributed) or Kruskal-Wallis test (abnormally distributed). Categorical variables were analyzed using the Pearson *χ*^2^ test or Fisher’s exact test as appropriate and were expressed as frequency (percentages). Patients were divided into subgroups of BMI < 24 kg/m^2^ group and BMI ≥ 24 kg/m^2^ group for stratified analysis. Multivariate logistic regression was used to assess the association between different types of lipid metabolism and cLBR by adjusting the effect of baseline characteristics and groups to calculate the odds ratios (ORs) and 95% confidence intervals (CIs). All values of *p* < 0.05 for two-sided tests were considered to be statistically significant.

## Results

A total of 2,963 patients underwent the ovarian stimulation and oocyte retrieval during the study period. Of these, 1831 (61.8%) patients were excluded due to any of the following reasons: older than 38 years old (323 patients, 10.9%), not the first stimulation cycle (1,091 patients, 36.8%), no embryo transferred by the time of data collection (288 patients, 9.7%), impaired ovarian reserve (105 patients, 3.5%), and incomplete records (24 patients, 0.8%). Therefore, 1,132 patients met the eligibility criteria, of whom 195 cases had dyslipidemia (17.2%) and 937 had normal lipid metabolism. The baseline characteristics are shown in Table 1. Patients with dyslipidemia had significantly greater BMI (23.77 ± 3.89 kg/m^2^ vs. 22.08 ± 3.61 kg/m^2^, *p* < 0.001) and longer duration of infertility (3.8 ± 2.1 years vs. 3.4 ± 2.4 years, *p* = 0.031) compared with patents without dyslipidemia. In addition, patients with dyslipidemia had significantly lower bilateral antral follicle count (17.38 ± 5.72 vs. 18.56 ± 6.29, *p* = 0.016) and basal FSH (6.28 ± 1.64 IU/L vs. 6.62 ± 1.72 IU/L, *p* = 0.012) than the control group. No differences were observed in terms of age, AMH, basal LH, basal FSH/LH ratio, basal E_2_, infertility type, causes of infertility, or ovarian stimulation protocol (*p* > 0.05).

**Table 1:** Baseline characteristics of non-PCOS patients with and without dyslipidemia.

Variables	Dyslipidemia group(*n* = 195)	Control group(*n* = 937)	Value of *p*
Age (y)	30.21 ± 3.34	30.02 ± 3.13	0.446
BMI (kg/m^2^)	23.77 ± 3.89	22.08 ± 3.61	<0.001
Duration of infertility (y)	3.8 ± 2.1	3.4 ± 2.4	0.031
AMH (ng/ml)	3.51 ± 1.57	3.77 ± 2.02	0.091
Bilateral AFC	17.38 ± 5.72	18.56 ± 6.29	0.016
Basal FSH (IU/L)	6.28 ± 1.64	6.62 ± 1.72	0.012
Basal LH (IU/L)	4.53 ± 2.01	4.90 ± 3.12	0.112
Basal FSH/LH ratio	1.73 ± 1.47	1.65 ± 1.36	0.461
Basal E_2_ (pg/mL)	41.33 ± 22.17	42.52 ± 24.64	0.533
Infertility type, *n* (%)			0.219^a^
Primary	83 (42.6)	444 (47.4)	
Secondary	112 (57.4)	493 (52.6)	
Cause of infertility, *n* (%)			0.577^b^
Tubal factor	111 (56.9)	559 (59.7)	
Male factor	62 (31.8)	284 (30.3)	
Mixed factors	19 (9.7)	88 (9.4)	
Unexplained	3 (1.6)	6 (0.6)	
Ovarian stimulation protocol			0.789^a^
GnRH agonist long	138 (70.8)	672 (71.7)	
GnRH antagonist	57 (29.2)	265 (28.3)	

*Data are presented as either means ± standard deviations or number (%). PCOS, polycystic ovary syndrome; BMI, body mass index; AMH, anti-Mullerian hormone; AFC, antral follicular count; FSH, follicle-stimulating hormone; LH, luteinizing hormone; E_2_, estradiol; and GnRH, gonadotropin-releasing hormone*.

a *Pearson χ^2^ test*.

b *Fisher’s exact test*.

Table 2 summarizes the cycle parameters and pregnancy outcomes of IVF/ICSI cycles. The initial dose of gonadotropins (155.04 ± 22.36 IU vs. 148.22 ± 31.44 IU, *p* = 0.004) and the total stimulation dose (1567.63 ± 532.30 IU vs. 1468.48 ± 430.16 IU, *p* = 0.005) used were significantly higher in the dyslipidemia group, although peak E_2_ (3522.88 ± 1989.47 vs. 3826.54 ± 1894.54, *p* = 0.044), the number of lead follicles on hCG day (10.69 ± 4.14 vs. 11.76 ± 3.99, *p* = 0.001), and number of oocytes retrieved (11.03 ± 5.63 vs. 12.52 ± 5.41, *p* = 0.001) were marked lower compared with patients in the non-dyslipidemia group. However, the fertilization rate, number of available embryos and embryos cryopreserved, the embryo utilization rate, proportion of fresh or frozen embryo transfer, and methods of endometrium preparation in FET cycles between two groups were comparable (*p* > 0.05).

**Table 2:** COH parameters and pregnancy outcomes in non-PCOS patients with and without dyslipidemia.

Variables	Dyslipidemia group(*n* = 195)	Control group(*n* = 937)	Value of *p*
Starting dose of Gn (IU)	155.04 ± 22.36	148.22 ± 31.44	0.004
Days of COH (d)	10.12 ± 1.45	9.94 ± 1.30	0.085
Total Gn dosage (IU)	1567.63 ± 532.30	1468.48 ± 430.16	0.005
Peak E_2_ on hCG day (pg/ml)	3522.88 ± 1989.47	3826.54 ± 1894.54	0.044
LH on hCG day (IU/L)	4.22 ± 3.61	4.14 ± 3.36	0.765
Progesterone on hCG day (ng/ml)	0.88 ± 0.39	0.87 ± 0.31	0.696
Number of ≥14 mm follicles on hCG day (*n*)	10.69 ± 4.14	11.76 ± 3.99	0.001
Number of oocytes retrieved (*n*)	11.03 ± 5.63	12.52 ± 5.41	0.001
Endometrial thickness on hCG day (mm)	11.44 ± 2.01	11.54 ± 2.07	0.538
Method of fertilization			0.907^a^
IVF, *n* (%)	137 (70.3)	673 (71.8)	
ICSI, *n* (%)	56 (28.7)	255 (27.2)	
IVF+ICSI, *n* (%)	2 (1.0)	9 (1.0)	
Fertilization rate (×100%)	0.69 ± 0.31	0.71 ± 0.24	0.316
Number of available embryos (*n*)	6.23 ± 3.05	6.62 ± 3.07	0.106
Number of embryos cryopreserved (*n*)	4.46 ± 2.55	4.83 ± 2.37	0.051
Embryo utilization rate, (%)	79.58	78.43	0.412
Fresh or frozen embryo transfer			0.943^b^
Fresh embryo transfer, *n* (%)	131 (67.2)	627 (66.9)	
FET, *n* (%)	64 (32.8)	310 (33.1)	
Methods of endometrium preparation in FET cycles			0.569^a^
HRT cycle *n* (%)	105 (50.5)	296 (50.4)	
Natural ovulation cycle, *n* (%)	33 (15.9)	85 (14.5)	
Downregulation cycle, *n* (%)	65 (31.2)	199 (33.9)	
Mild stimulation cycle, *n* (%)	5 (2.4)	7 (1.2)	
Stage of embryo transferred			0.491^a^
Cleavage-stage transfer	188	673	
Blastocyst transfer	158	615	
Mean number of ET	1.82 ± 0.55	1.81 ± 0.65	0.841
Moderate-severe OHSS^c^ *n* (%)	8 (6.1)	15 (2.4)	0.043^a^
Clinical pregnancy rate^c^, *n* (%)	75/131 (57.3)	378/627 (60.3)	0.519^b^
Miscarriage rate^c^, *n* (%)	13/75 (17.3)	34/378 (9.0)	0.031^b^
Live-birth rate^c^, *n* (%)	62/131 (47.3)	344/627 (54.9)	0.116^b^
Twin delivery	6/131 (4.6)	26/627 (4.1)	0.793
Cumulative live-birth rate, *n* (%)	121/195 (62.1)	633/937 (67.6)	0.138^b^
Pregnancy complications			
Gestational diabetes	5 (2.6)	14 (1.5)	0.352^a^
Preeclampsia	1 (0.5)	12 (1.3)	0.709^a^
Birth weight (g)			0.388
Singleton	3,114 ± 603	3,110 ± 552	0.443
Twin	3,075 ± 260	2,715 ± 431	0.517

*Data are presented as either means ± standard deviations or number (%). COH, controlled ovarian hyperstimulation; PCOS, polycystic ovary syndrome; Gn, gonadotrophin; E_2_, estradiol; hCG, human chorionic gonadotropin; LH, luteinizing hormone; IVF, in vitro fertilization; ICSI, intracytoplasmic sperm injection; FET, frozen embryo transfer; HRT, hormone replacement treatment; ET, embryo transfer; and OHSS, ovary hyperstimulation syndrome*.

a *Fisher’s exact test*.

b *Pearson χ^2^ test*.

c *The rate in fresh embryo transfer cycle*.

Notably, there was a significantly higher incidence of moderate-severe OHSS in the dyslipidemia group compared with the normal group, as shown in Table 2 (6.1% vs. 2.4%, *p* = 0.043). Similarly, women with dyslipidemia had a higher chance of miscarriage compared with the women in the normal group (17.3% vs. 9.0%, *p* = 0.031).

The live-birth rate in fresh cycle and cLBR were higher in non-dyslipidemia group although the difference between the groups was not significant (54.9% vs. 47.3%, *p* = 0.116; 62.1% vs. 67.6%, *p* = 0.138). Other outcomes, including the rates of clinical pregnancy and twin birth, and pregnancy-related complications, including gestational diabetes, preeclampsia, and birth weights, showed no differences between the groups (*p* > 0.05).

To further determine whether the effect of dyslipidemia on reproductive outcomes was independent of obesity, patients were stratified based on their BMI (kg/m^2^) as normal (BMI < 24 kg/m^2^) and overweight/obese (BMI ≥ 24 kg/m^2^), as shown in Table 3. We found that dyslipidemia was associated with significantly higher total dose of Gn and lower number of oocytes retrieved, independent of obesity. Furthermore, overweight/obese women with dyslipidemia required a significantly higher starting dose of Gn, but the difference was no longer significant in normal weight group. It is worth noting that the incidence rate of moderate-severe OHSS in the BMI ≥ 24 kg/m^2^ women with dyslipidemia was higher than other groups, although the difference was not significant, which may be related to the more dosage and days of Gn.

**Table 3:** Subgroup analysis of main parameters in different BMI groups with and without dyslipidemia.

Variables	BMI < 24 kg/m^2^			BMI ≥ 24 kg/m^2^		
	Dyslipidemia group(*n* = 62)	Control group(*n* = 733)	Value of *p*	Dyslipidemia group(*n* = 133)	Control group(*n* = 204)	Value of *p*
Starting dose of Gn (IU)	146.21 ± 23.44	143.36 ± 27.21	0.424	162.07 ± 32.48	153.11 ± 42.22	0.046
Days of COH (d)	10.20 ± 1.33	9.85 ± 2.13	0.204	11.09 ± 2.16	10.82 ± 1.67	0.198
Total Gn dosage (IU)	1561.26 ± 557.03	1435.22 ± 522.62	0.031	1623.35 ± 504.81	1504.63 ± 512.59	0.037
Peak E_2_ on hCG day (pg/ml)	3316.31 ± 1327.04	3627.47 ± 1681.64	0.156	3622.88 ± 2012.15	3811.67 ± 1863.27	0.379
Number of ≥14 mm follicles on hCG day (*n*)	11.62 ± 4.21	12.05 ± 3.48	0.193	9.98 ± 3.91	10.13 ± 3.66	0.721
Number of oocytes retrieved (*n*)	12.21 ± 4.33	13.77 ± 5.03	0.018	10.01 ± 5.09	11.26 ± 5.21	0.031
Moderate-severe OHSS^b^ *n* (%)	1(1.1)	5(1.7)	0.226^c^	7(5.3)	10(4.9)	0.277^c^
Endometrial thickness on hCG day (mm)	11.31 ± 2.42	11.67 ± 2.58	0.290	11.71 ± 2.22	11.37 ± 2.01	0.146
Fertilization rate (×100%)	0.72 ± 0.28	0.74 ± 0.35	0.661	0.67 ± 0.27	0.68 ± 0.15	0.661
Number of available embryos (*n*)	6.55 ± 3.14	6.77 ± 2.98	0.578	6.16 ± 3.01	6.42 ± 3.03	0.441
Cumulative live-birth rates, *n* (%)	39/62 (62.9)	501/733 (68.3)	0.378^a^	82/133 (61.7)	132/204 (64.7)	0.570^a^

*Data are presented as either means ± standard deviations or number (%). BMI, body mass index; Gn, gonadotrophin; COH, controlled ovarian hyperstimulation; E2, estradiol; hCG, human chorionic gonadotropin; and LH, luteinizing hormone*.

a *Pearson χ^2^ test*.

b *The rate in fresh embryo transfer cycle*.

c *Fisher’s exact test*.

Univariate logistic regression was first performed to evaluate the effect of each variable on cLBR (Table 4). Thereafter, variables with a value of *p* < 0.1 and important known potential confounders were included in the final multivariable logistic regression model by manual forward selection. Table 4 shows there is a significant decrease in the cLBR in dyslipidemia women when compared with women with normal lipid metabolism (OR, 0.698, 95% CI, 0.531–0.878, *p* = 0.033). There was a positive correlation between the number of oocytes retrieved, available embryos, and embryos cryopreserved on the cLBR (OR, 2.226, 95% CI, 1.095–4.101, *p* = 0.018; OR, 2.981, 95% CI, 1.685–4.271, *p* < 0.001; OR, 3.184, 95% CI, 2.377–5.441, *p* < 0.001).

**Table 4:** Results of univariate analysis for cLBR.

Variables	OR (95% CI)	Value of *p*
Age	0.962 (0.891–1.011)	0.651
BMI	0.896 (0.822–0.953)	0.041
Infertility duration	0.933 (0.866–1.041)	0.144
AMH	1.033 (0.991–1.040)	0.832
Bilateral AFC	0.971(0.912–1.015)	0.316
Basal FSH	0.977 (0.891–1.011)	0.891
Basal LH	0.988 (0.976–1.072)	0.974
Basal FSH/LH ratio	1.000 (0.996–1.013)	0.982
Basal E_2_	1.021 (0.891–1.033)	0.533
**Lipid metabolism**		
Non-dyslipidemia	1	
Dyslipidemia	0.698 (0.531–0.878)	0.033
**Infertility type**		
Primary	1	
Secondary	0.942 (0.843–1.019)	0.082
**Ovarian stimulation protocol**		
GnRH agonist long	1	
GnRH antagonist	0.941 (0.644–1.231)	0.743
Total Gn dosage	0.865 (0.588–1.291)	0.463
Peak E2 on hCG day	1.371 (0.462–3.575)	0.646
Number of oocytes retrieved	2.226 (1.095–4.101)	0.018
**Method of fertilization**		
IVF	1	
ICSI	1.077 (0.943–2.175)	0.108
IVF+ICSI	1.241 (0.554–3.663)	0.309
Fertilization rate	1.145 (0.763–2.624)	0.721
Number of available embryos	2.981 (1.685–4.271)	<0.001
Number of embryos cryopreserved	3.184 (2.377–5.441)	<0.001
**Stage of embryo transferred**		
Cleavage-stage transfer	1	
Blastocyst transfer	1.008 (0.899–1.029)	0.726
Mean number of ET	0.789 (0.426–1.396)	0.357
**Moderate-severe OHSS**		
No	1	
Yes	1.139 (0.582–2.429)	0.781
**Methods of endometrium preparation in FET cycles**		
HRT cycle	1	
Natural ovulation cycle	1.014 (0.921–1.032)	0.729
Downregulation cycle	1.011 (0.883–1.022)	0.718
Mild stimulation cycle	1.022 (0.962–1.041)	0.916

*cLBR, cumulative live-birth rate; OR, odds ratio; CI, confidence interval; BMI, body mass index; AMH, anti-Mullerian hormone; AFC, antral follicular count; FSH, follicle-stimulating hormone; LH, luteinizing hormone; E_2_, estradiol; GnRH, gonadotropin-releasing hormone; Gn, gonadotrophin; hCG, human chorionic gonadotropin; IVF, in vitro fertilization; ICSI, intracytoplasmic sperm injection; ET, embryo transfer; OHSS, ovary hyperstimulation syndrome; FET, frozen embryo transfer; and HRT, hormone replacement treatment*.

After adjusting for age, BMI, AMH, infertility type, total Gn dosage, number of oocytes retrieved, fertilization rate, number of available embryos, and number of embryos cryopreserved by multivariate logistic regression analysis, dyslipidemia was still significantly associated with decreased cLBR (OR, 0.702, 95% CI, 0.533–0.881, *p* = 0.044; Table 5). Moreover, the numbers of oocytes retrieved, available embryos, and embryos cryopreserved were positively associated with cLBR (OR, 2.217, 95% CI, 1.104–4.122, *p* = 0.017; OR, 2.787, 95% CI, 1.691–4.283, *p* < 0.001; OR, 3.279, 95% CI, 2.353–5.432, *p* < 0.001; Table 5).

**Table 5:** Outcomes of multivariate logistic analysis of the impact of dyslipidemia on cLBR.

Variables	Adjusted OR (95% CI)	Value of *p*
Age	1.174 (0.822–1.281)	0.562
BMI	0.728 (0.504–1.053)	0.072
AMH	0.882 (0.521–1.039)	0.094
**Infertility type**		
Primary	1	
Secondary	1.273 (0.442–3.584)	0.649
Total Gn dosage	0.905 (0.476–1.677)	0.566
Number of oocytes retrieved	2.217 (1.104–4.122)	0.017
Fertilization rate	1.167 (0.721–2.632)	0.682
Number of available embryos	2.787 (1.691–4.283)	<0.001
Number of embryos cryopreserved	3.279 (2.353–5.432)	<0.001
**Lipid metabolism**		
Non-dyslipidemia	1	
Dyslipidemia	0.702 (0.533–0.881)	0.044

*cLBR, cumulative live-birth rate; OR, odds ratio; CI, confidence interval; BMI, body mass index; AMH, anti-Mullerian hormone; and Gn, gonadotrophin*.

## Discussion

To the best of our knowledge, this is the first large cohort study to investigate the relationship between clinical dyslipidemia and IVF/ICSI outcomes, particularly the rates of cumulative live-birth in the general population. The prevalence of dyslipidemia in our study (17.2%) was lower than the average incidence rate (40.7%) in the general population of China aged 18 and older (Joint Committee for Guideline Revision, 2018), due to the exclusion of patients with PCOS. Despite this, it is still necessary to explore the impact of dyslipidemia on ART results when considering the large number of infertile people.

In our study, we found that patients with dyslipidemia had longer duration of infertility, and lower AFC and basal FSH levels. Notably, subgroup analysis demonstrated that dyslipidemia was associated with significantly higher total Gn dosage required for ovarian stimulation and lower number of oocytes retrieved, independent of obesity. In addition, there was a higher incidence of OHSS and miscarriage in patients with dyslipidemia compared to those with normal lipid metabolism. The live-birth rate in the fresh cycle and the cLBR were higher in the non-dyslipidemia group, although the difference was not significant. Multivariate logistic regression model showed that dyslipidemia alone is negatively associated with cLBR in non-PCOS patients.

Previous studies have shown that dyslipidemia is positively correlated with body weight, and patients with dyslipidemia are more prone to obesity. These findings are consistent with results from our study in which patients with dyslipidemia were associated with significantly greater BMI, suggesting that obesity and dyslipidemia have a close relationship. We also found that patients with dyslipidemia required higher initial and total gonadotropin doses compared to patients with normal lipid metabolism during COH protocol. This is because the dose of gonadotropin required is usually adjusted by physicians based on the BMI of the patients. In other words, patients with higher BMI require a higher dose of gonadotropin to promote follicle development. Our findings on OHSS are corroborated by a recent study in which patients with dyslipidemia were reported to be more likely to develop severe OHSS in the freeze-all embryos group (Liu et al., 2020). There are studies that have shown that dyslipidemia, which is characterized by elevated plasma TG and LDL levels, causes damage to the vascular wall (Jenkins et al., 2004; Urbina et al., 2017). Moreover, it is generally accepted that high cholesterol levels can also adversely affect the microvasculature prior to its inducing of inflammation (Padro et al., 2018). In addition, BMI and the total dose of gonadotropin required are considered to be the main risk factor affecting the development of OHSS. This explains the higher incidence of OHSS observed in patients with dyslipidemia compared to those with normal lipid metabolism in our study.

It is well known that disordered lipid metabolism usually occurs due to changes in lifestyle, including physical inactivity and excessive intake of *high*-*fat* diet (HFD). Physiologically, lipids are important for energy production during oocyte maturation, fertilization, and preimplantation development. Studies using mice models have shown that HFD causes lipotoxicity in granulosa and cumulus cells, resulting in accelerated ovarian follicle development and follicle loss, which eventually affects ovarian reserve (Wu et al., 2010). Likewise, Skaznik-Wikiel et al. reported that HFD causes significant reduction in ovarian reserve, excessive production of pro-inflammatory cytokines, and compromised fertility in rodents, regardless of the obesity phenotype (Skaznik-Wikiel et al., 2016). These findings in animal models suggest that obesity has a lesser impact on ovarian function compared to HFD (Hohos and Skaznik-Wikiel, 2017). Similarly, results from our study showed a significant association between dyslipidemia and decreased AFC as well as the number of oocytes retrieved compared to the healthy group. Further studies are required to assess these findings.

Moreover, a recent study found that the patients with accumulation of lipid droplets in cumulus and granulosa cells had lower IVF/ICSI pregnancy rates, regardless of their BMI (Raviv et al., 2020). Excess fatty acids could damage the cumulus and granulosa cells, which are the major functional cells of the female gonads thus impairing their ability to perform normal steroidogenesis (Raviv et al., 2020). High dietary fat intake, with or without the development of obesity, impairs female hypothalamic-pituitary-ovarian (HPO) axis as well as fertility (Hohos and Skaznik-Wikiel, 2017). Mice on HFD were found to have mitochondrial dysfunction, which can lead to serious defects and chromosomal dislocation in meiosis of oocytes. This causes a decline in the developmental potential of embryos and the loss of early embryos (Luzzo et al., 2012; Grindler and Moley, 2013). Findings from these studies may explain why patients with abnormal lipid profiles in our study were associated with significantly lower basal FSH and peak estradiol levels as well as higher abortion rates compared to patients with normal lipid profiles.

The cumulative delivery rate was mainly affected by the age of patients and the number of oocytes retrieved during the COS process (Polyzos et al., 2018). Recently, Wang et al. demonstrated that serum levels of TG, TC, and LDL were negatively correlated, while HDL and Lp (a) levels were positively correlated with the embryo quality. Results from previous studies as well as the findings from our study suggest that dyslipidemia is negatively correlated with cumulative delivery rates in IVF/ICSI cycles due to its effect on the number of oocytes retrieved and the quality of embryos. This may be attributed to the higher dosage of Gn required as well as the persistent lipotoxicity associated with dyslipidemia patients causing a decline in the number of oocytes retrieved, the compromised embryonic development, and impaired endometrial receptivity, regardless of the obesity phenotype. More research is required to identify the potential mechanisms.

There are several strengths in this study, including the large sample size from a relatively homogenized cohort of people. In addition, we carried out subgroup analysis to eliminate the effect of obesity on the IVF/ICSI outcomes. Lastly, our study excluded the effect of PCOS, a common endocrine disorder contributing to subfertility, on reproductive outcomes. On the other hand, there are several limitations in our study. First, we could not investigate unknown confounding factors, such as dietary habits or medical interventions before or during IVF treatment due to the retrospective nature of the study. Secondly, the association between individual lipoproteins and IVF/ICSI outcomes could not be determined due to the sample size. Thirdly, while our results are convincing, they should be taken with caution since only the results of multivariate logistic analysis were used to conclude that dyslipidemia has detrimental effects on cLBR. Further longitudinal investigations and larger multicenter clinical investigations are required to validate these results and to explore the mechanism behind these effects.

## Conclusion

In conclusion, our findings demonstrate for the first time that dyslipidemia has a deleterious impact on cLBR, independent of obesity, in non-PCOS population considered to have good prognosis. Assessment of serum lipid profiles as well as the provision of nutritional counseling is essential for increasing successful outcomes in assisted reproductive techniques.

## Data Availability Statement

The raw data supporting the conclusions of this article will be made available by the authors, without undue reservation.

## Ethics Statement

The studies involving human participants were reviewed and approved by the Ethics Committee of Yantai Yuhuangding Hospital. Written informed consent for participation was not required for this study in accordance with the national legislation and the institutional requirements.

## Author Contributions

ZL and JC conceived and designed the study. ZL and XL took part in the clinical data collection and analyzed the data. ZL, HZ, SL, and SH wrote the manuscript. JC and HB supervised the entire work. All authors contributed to the article and approved the submitted version.

## Conflict of Interest

The authors declare that the research was conducted in the absence of any commercial or financial relationships that could be construed as a potential conflict of interest.

## Publisher’s Note

All claims expressed in this article are solely those of the authors and do not necessarily represent those of their affiliated organizations, or those of the publisher, the editors and the reviewers. Any product that may be evaluated in this article, or claim that may be made by its manufacturer, is not guaranteed or endorsed by the publisher.
